# Biomimetic propulsion system efficiency for unmanned underwater vehicle

**DOI:** 10.1038/s41598-025-95702-7

**Published:** 2025-04-01

**Authors:** Piotr Szymak, Paweł Piskur, Rafał Kot, Krzysztof Naus, Daniel Powarzyński

**Affiliations:** Polish Naval Academy, Smidowicza 69, 81-127 Gdynia, Poland

**Keywords:** Mechanical engineering, Biomimetics

## Abstract

This paper covers experimental research provided for Biomimetic Unmanned Underwater Vehicle (BUUV). The tests were conducted in a laboratory water tunnel equipped with a direct force-measured sensor and system for Particle Image Velocimetry (PIV) analysis. Different control parameters were tested, and then the generated thrust was compared with electric energy consumption. The main goal of the research is to develop a low hydroacoustic noise and high-energy efficiency propulsion system based on single, flexible fins. The final result is a set of Pareto optimal solutions, which makes it possible to draw more general conclusions on the design of the undulating propulsion system.

## Introduction

Electromagnetic waves are strongly attenuated in water, therefore, the main source of passive detection of an underwater vehicle is hydroacoustic waves generated by the propulsion system^1^. One of essential advantages of biomimetic propulsion systems is the low hydroacoustic spectrum^2^, which makes it able to covertly observe flora and fauna as well as make inspections of underwater infrastructure without disruption^3^.

**Fig. 1:**
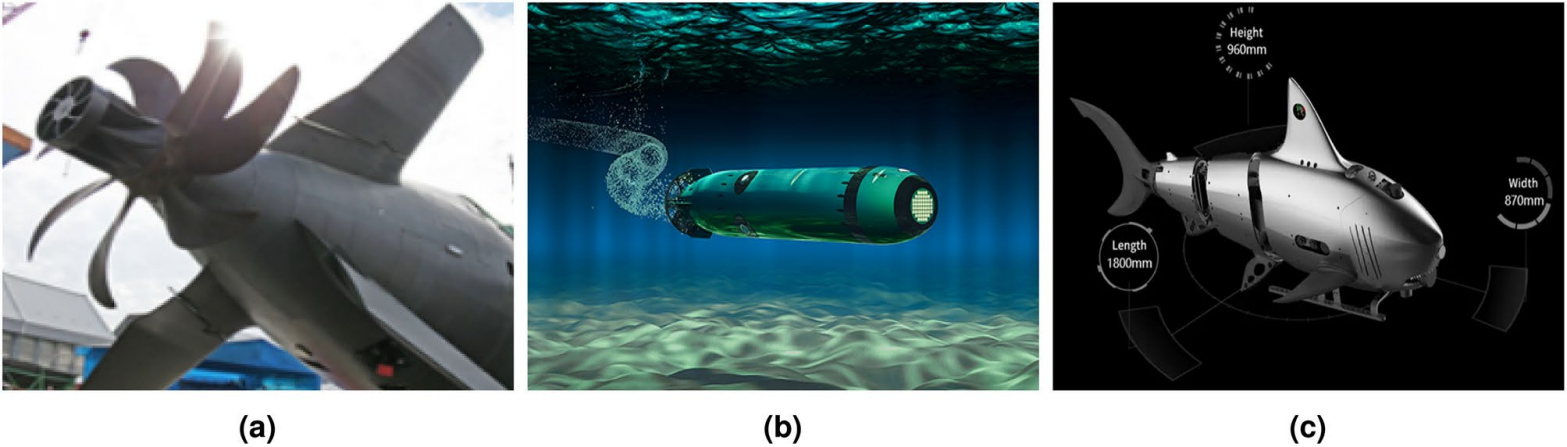
Submarine propeller (**a**), propeller-driven unmanned underwater vehicle (**b**), biomimetic underwater vehicle made from connected rigid parts (**c**) source: https://www.curtisswrightds.com/resources/case-studies/uuv-nas-protects-terabytes-top-secret-mission-data.

Research is ongoing in this field to develop more advanced and efficient designs that can be used for various applications, including environmental monitoring, oceanography, and underwater exploration^4^. The biomimetic propulsion system generates a hydroacoustic signature that is more difficult to detect compared to ship propellers (Fig. 1a) used in underwater vehicles (Fig. 1b). One of the biomimetic drive structures used, consisting of rigid elements connected together, is shown in Fig. 1c.

Underwater vehicles can be constructed in various ways depending on the desired use and environment^5^. Their design depends on the shape, e.g. torpedo shapes^1^, manta ray, harbour seal^6^ and the number of propulsors, mainly propellers^7^. Biomimetic Unmanned Underwater Vehicle (BUUV)—imitate biological organisms that evolved through natural selection over many years^8^, imitating a fish-like movement^9^, a turtle^10^, a seal^11^ or other marine animals.

**Fig. 2:**
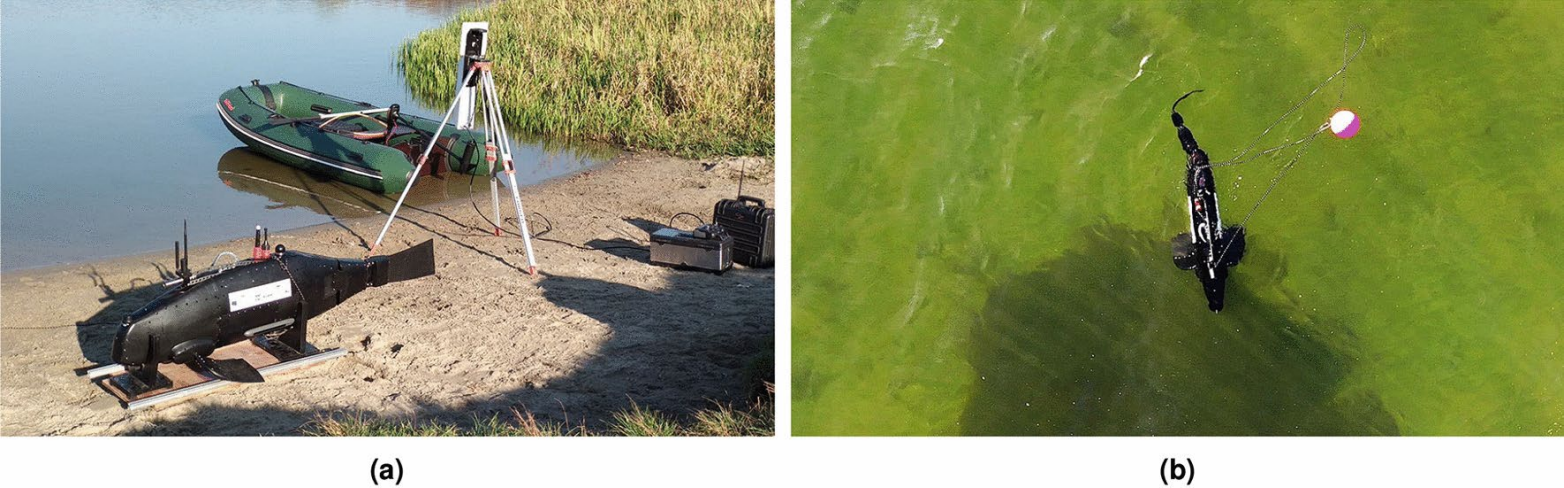
Biomimetic unmanned underwater vehicle: (**a**) on the shore of the lake, (**b**) during tests in the Bay of Gdańsk.

The biomimetic, unmanned underwater vehicle presented in Fig. 2 was developed by scientists from the Cracov University of Technology as part of the SABUVIS (Swarm of Biomimetic Unmanned Underwater Vehicles for Underwater ISR) project in cooperation with the Polish Naval Academy, financed by the European Defense Agency. The drive consists of several rigid parts, and the last flexible one. Biomimetic multi-unit drives are used to reproduce the kinematics of a fishtail. However, the multi-joint unit requires several actuators, which increase construction and control costs, limit the space inside the vehicle hull for sensors and power systems, and make the structure more expensive and more susceptible to leaks.

The vehicles developed during the same project (SABUVIS) at the Polish Naval Academy use single-piece flexible fins in two configurations. A single-fin drive used for unmanned underwater vehicles is a cheaper solution to build and operate than a multi-body drive. Fewer moving connections increase the reliability of the drive and reduce the likelihood of leaks and flooding of electronic components located inside the hull. Another advantage of the biomimetic single-fin drive is that it provides more space inside the vehicle than a multi-fin drive. This space can be used for additional power sources and sensors for various purposes. It is also possible to reduce the dimensions of the vehicle’s hydrodynamic resistance and increase the mission’s range and duration.

**Fig. 3:**
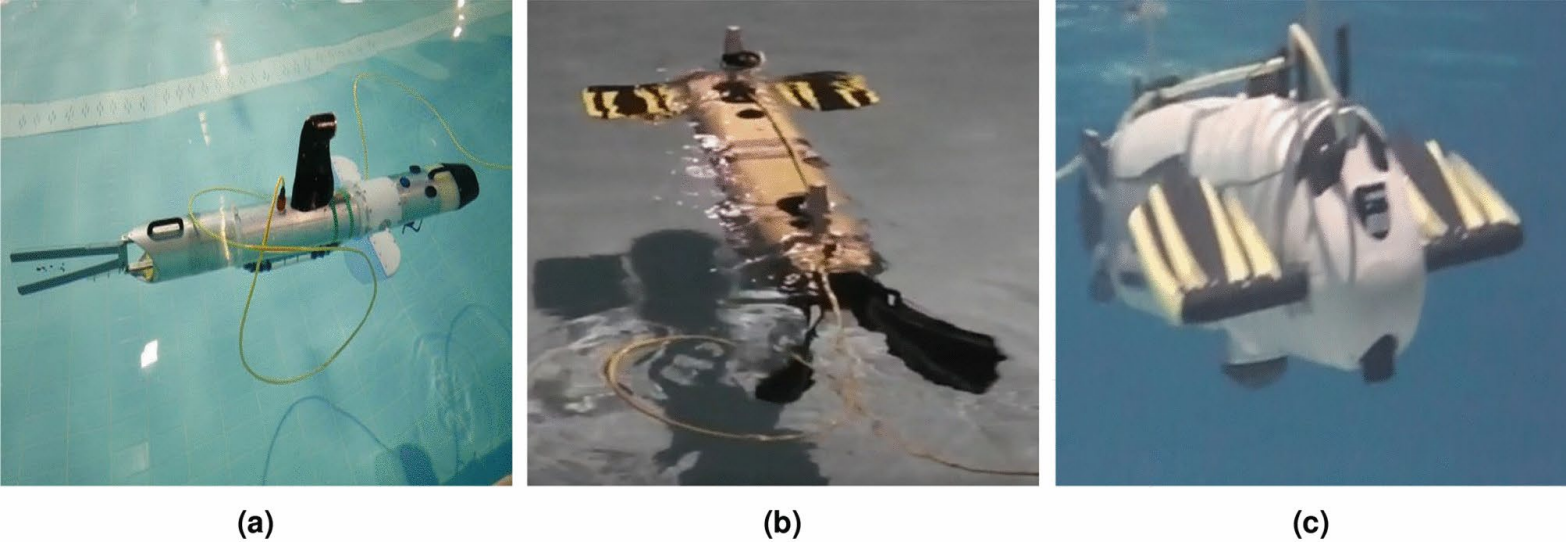
Biomimetic unmanned underwater vehicles: with one flexible tail fin and two side (**a**), with two tail fins and two side fins: stern view (**b**), bow view (**c**).

Figure 3a shows an underwater vehicle with a single-element, flexible tail drive, while Fig. 3b,c show an unmanned underwater vehicle with a propulsion system consisting of two flexible tail fins. All presented biomimetic underwater vehicles additionally had two side fins. The name biomimetic fins is dictated by the adopted assumptions of faithful reproduction in terms of controlling and designing living marine organisms.

One of the main goals of this research is to obtain a simpler structure solution of the propulsion system, therefore more reliable and at the same time of high energy efficiency. The detailed goal of the work is to develop a single fin with appropriate control parameters and find out the relations between control parameters, generating thrust and, as a result, the energy efficiency ratio.

The next sections include a literature analysis, a mathematical description of the propulsion system efficiency, and the laboratory water tunnel and measurement method used. Further, the results are presented based on direct thrust net and electric energy consumption measurements. The findings are discussed at the end of this study, and the methodology outline is proposed.

## State of the art

An energy-efficient underwater vehicle aims to minimize energy consumption while maximizing its operational capabilities. This is achieved through various means such as using electric or hybrid propulsion systems, energy-efficient batteries or other energy storage systems^12^, optimized hydrodynamic design, and the use of renewable energy sources^13^.

The interaction of the biomimetic flexible fins and fluid depends on environmental parameters (describing the properties of the fluid), material and geometric parameters (describing the shape of the fins), kinematic parameters (describing the movement of the fins) and parameters of the actuator system. When analyzing the energy efficiency of propulsion systems, the designing process can be seen as challenging, mainly because many non-linear phenomena need to be taken into account. The analysis presented in^14^ revealed the propulsive efficiency differences between many fish species (bluegill sunfish, rainbow trout, swordfish, tuna and shark). Based on the fish analysis, the desired range of the Strouhal number is between 0.2 and 0.4^15^. The Strouhal number includes the frequency of fin movements, the amplitude of the fin trailing edge and the fluid velocity. For artificial flexible fins, the amplitude of the trailing edge depends on the material parameters of the fin, their shapes, control parameters and vehicle speed. The mutual influence can be analysed using Buckingham’s theory^16^. This theory makes it possible to express all the information contained in the interactions between physical variables concisely, using a reduced number of dimensionless variables, as presented in the paper^17^. The material parameters’ impact on the propulsion system performance is depicted in the paper^18^, while in the paper^19^, the fin dimensions impact on the thrust net generation is analysed. When the vehicle velocity changes, the frequency of fin movement also needs to change to maintain the desired range of the Strouhal number. The efficiency of an artificial fish’s propulsion system is a trade-off between various design considerations and can be optimised for specific applications and environments.

**Fig. 4:**
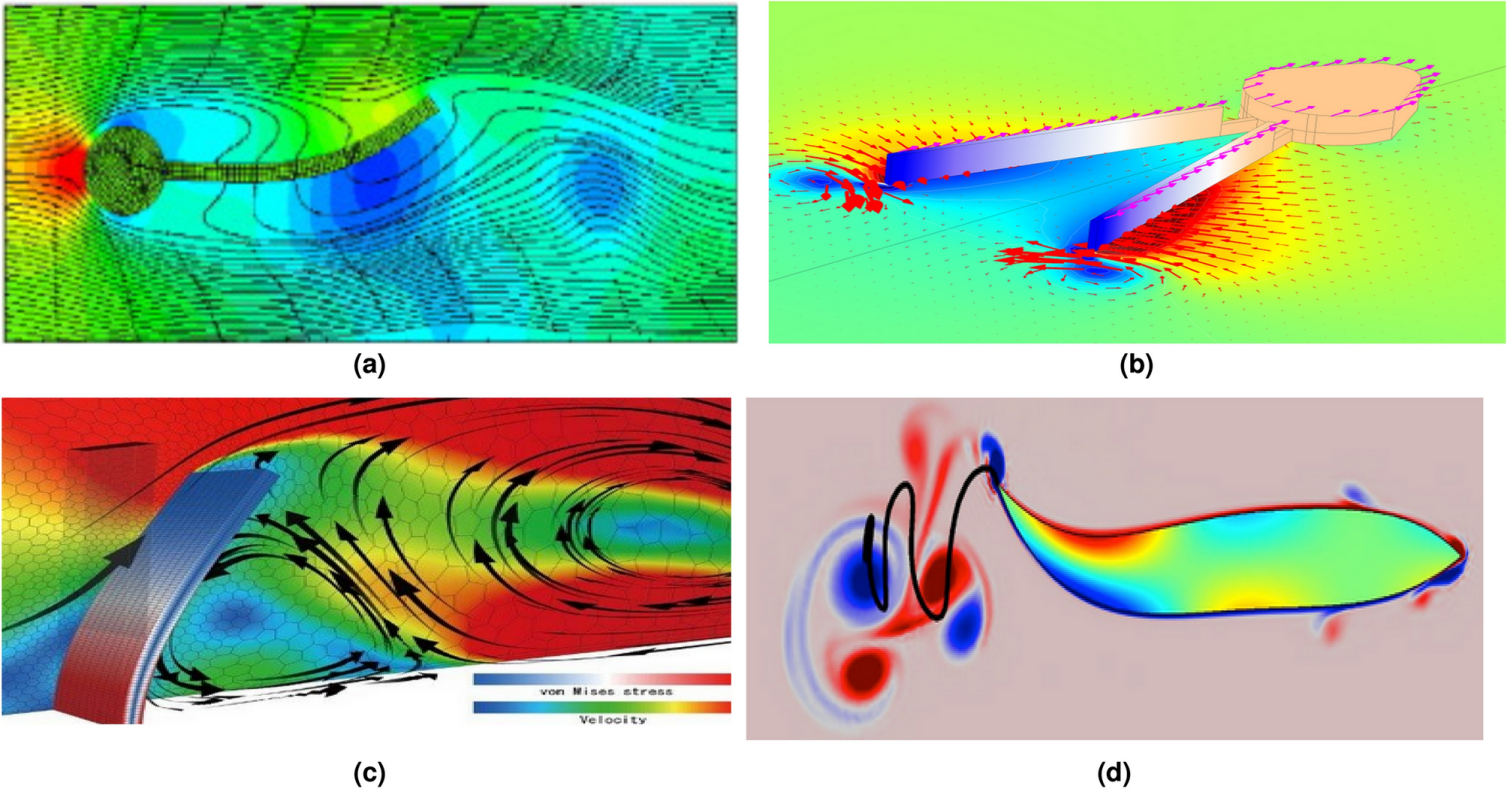
Fluid-Stucture Interaction: (**a**), (**c**) the effect of a viscous fluid on a flexible profile; (**b**), (**d**) the impact of rigid profiles on a viscous fluid (**a**) source: https://www.sciencedirect.com/science/article/pii/S0021999113007237 (**b**) source: https://www.comsol.de/blogs/modeling-fluid-structure-interaction-in-multibody-mechanisms/ (**c**) source: https://www.eostrack.com/blog/fluid-structure-interaction-apracticalperspective (**d**) source: https://www.comsol.com/blogs/studying-the-swimming-patterns-of-fish-with-simulation/.

Based on the literature analysis, it can be concluded that simulation models are available that describe the impact of a viscous fluid on a flexible profile fixed at one end (Fig. 4a,c). An example of the analysis of the impact of a rigid body on a viscous fluid are the models shown in Fig. 4b,d. The drive shown in Fig. 4b consists of two rigid fins. Also, the fish model in Fig. 4d is modelled as a rigid body with time-varying geometry.

**Fig. 5:**
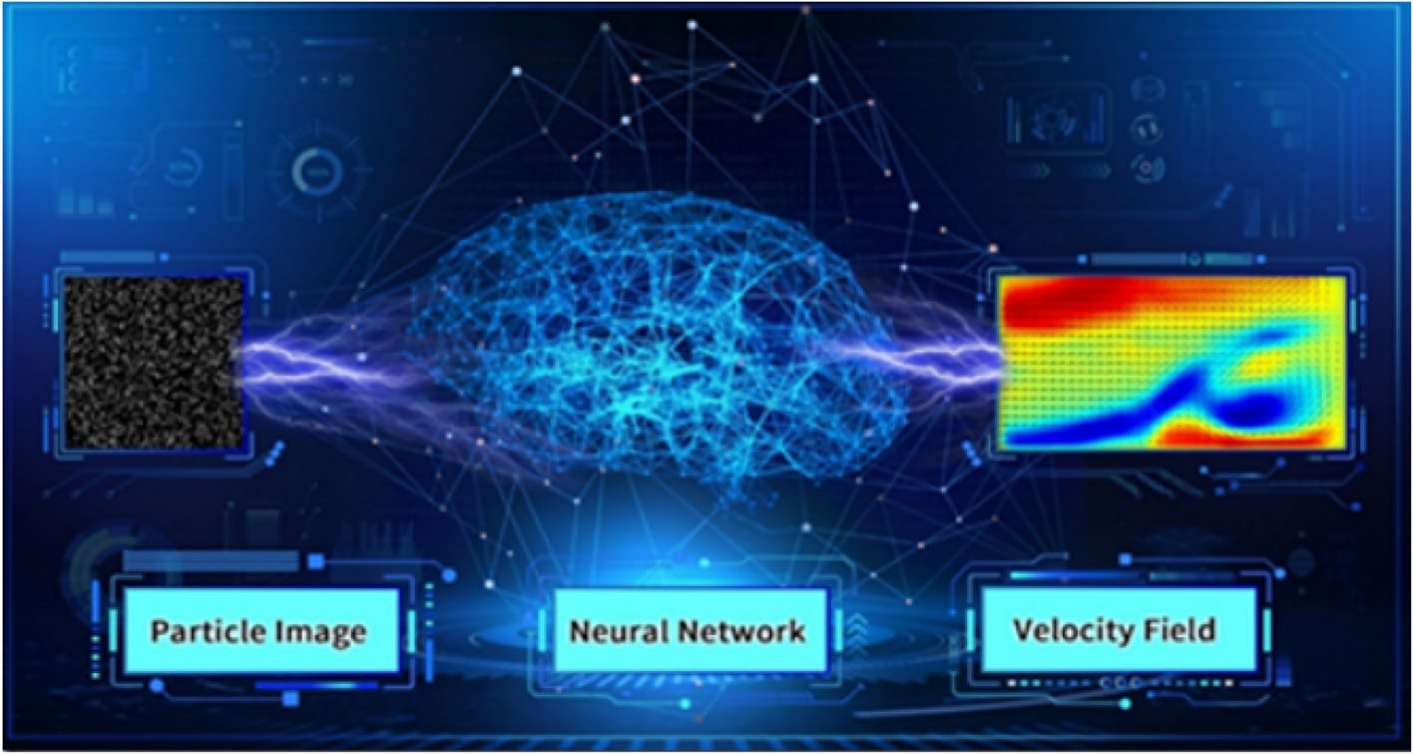
Neural network training scheme applied to fluid mechanics source: https://hsi.ca/product/ai-piv/.

However, the complexity of fluid turbulence remains a challenge in FSI, leading into the application of artificial neural networks for analysis and optimization. The training framework for these networks, as illustrated in Fig. 5, relies heavily on experimental data acquired from water tunnel tests. Recent advancements in artificial intelligence (AI) improves the modelling and optimization of FSI. Deep learning techniques have been effectively employed to enhance the accuracy of Particle Image Velocimetry (PIV) measurements, facilitating noise reduction, super-resolution reconstruction of velocity fields, and data interpolation^20^. Furthermore, the integration of evolutionary algorithms, such as Particle Swarm Optimization (PSO), has enabled real-time adaptation of propulsion control parameters, leading to a reported 4.5-fold increase in efficiency compared to conventional optimization approaches^21^. The incorporation of Physics-Informed Neural Networks (PINNs) has further enhanced numerical simulations by solving governing differential equations directly from sparse measurement data, refining computational fluid dynamics (CFD) models and reducing the dependency on high-fidelity simulations^22^. In addition, reinforcement learning (RL)-based adaptive control strategies have facilitated real-time optimization of biomimetic propulsion mechanisms, dynamically adjusting operational parameters to fluctuating hydrodynamic conditions, and improving maneuverability and energy efficiency in autonomous underwater vehicles (AUVs)^21^. The development of hybrid AI-CFD models has further accelerated the iterative design process, reducing computational costs while optimizing wave-based propulsion systems^23^. These transformative potential offering novel methodologies for enhancing fluid-structure interaction efficiency, energy savings, and operational performance in next-generation autonomous marine systems.

Further research was conducted in water tunnels, taking into account the greater reliability of the results from experimental tests carried out in water tunnels compared to simulation tests. The Particle Image Velocimetry (PIV) method was used to analyze fluid-structure interaction (FSI) phenomena in detail. Details related to the laboratory stand and measurement methods are described in the next section.

## Laboratory test equipment and measurement methods

**Fig. 6:**
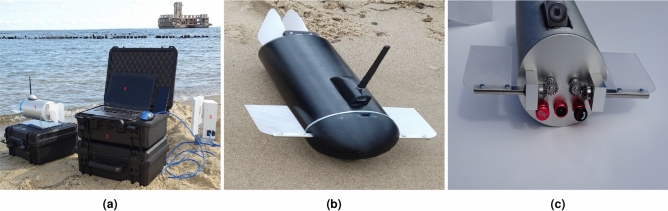
The BUUV before the test in a sea with control station and WiFi module (**a**), the BUUV on a sandy beach (**b**), and front view (without a bow dome) of the propeller shaft with bevel gear (**c**).

The BUUV presented in Fig. 6 is a smaller version of the vehicle from Fig. 3, without an expensive navigation system^24^. It was made by a team of students from the Polish Naval Academy during the Ministry of Defence competition. Also, the dimensions are more adequate for laboratory tests as well^25^.

**Fig. 7:**
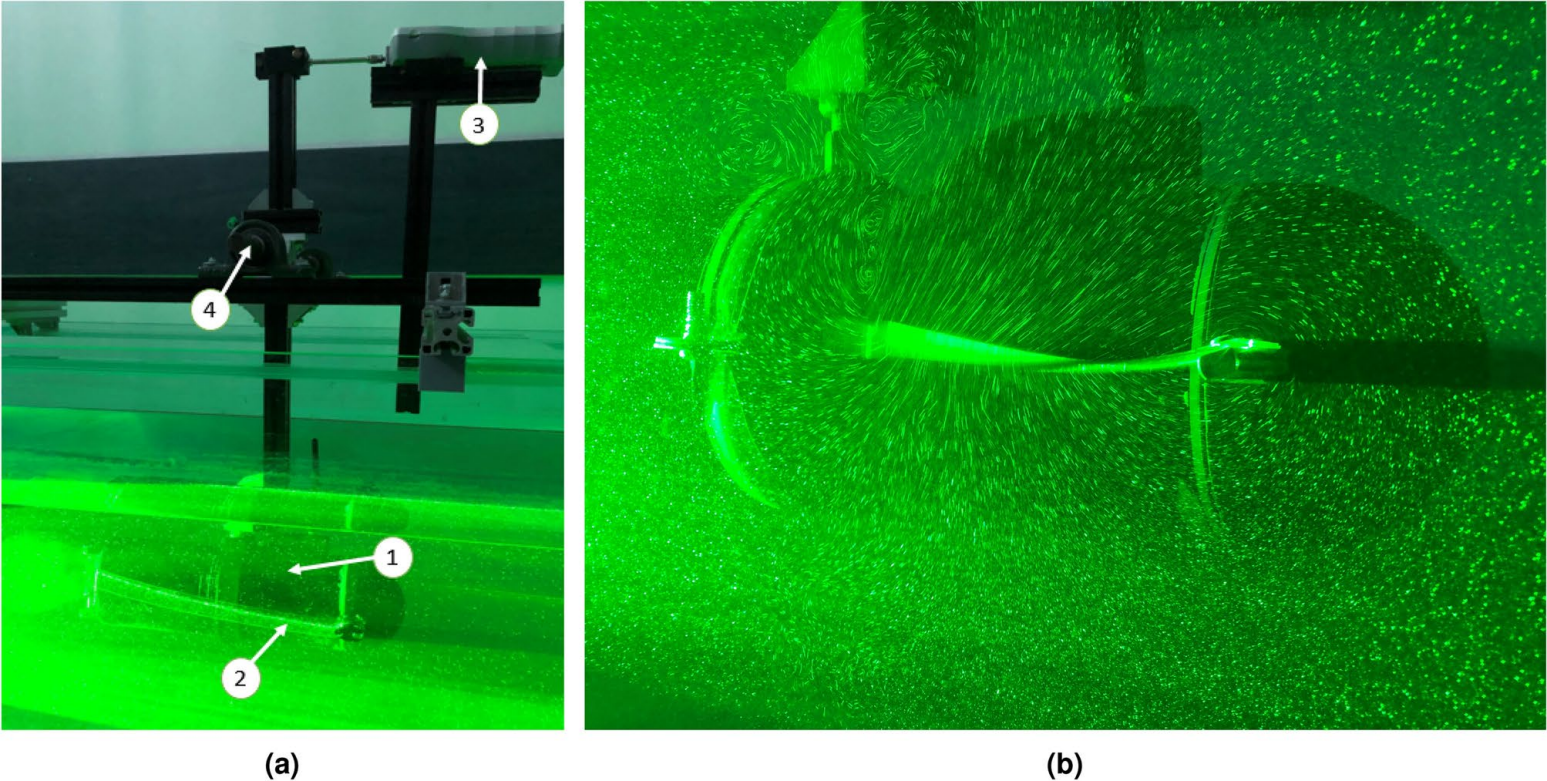
The laboratory water tunnel and green linear laser highlighting one of the side fin (**a**): 1–BUUV, 2–the side fin, 3–the force sensor, and 4–a ball bearing; (**b**) particles illuminated by a linear green laser.

The BUUV was used for tests in a laboratory water tunnel with dimensions of 0.5 m in width and 0.5 m in depth to work on the control parameters of the propulsion system. The water in the tunnel was maintained at temperature 20°C and atmospheric pressure, with a dynamic viscosity of approximately 1.002 mPa·s, ensuring realistic experimental conditions for evaluating propulsion performance. The water tunnel presented in Fig. 7 was designed especially for the biomimetic propulsion system investigation concerning BUUV dimensions. The walls were made from glass to provide laser illumination from different places. Thanks to the fin material’s transparency, the fin can be illuminated with a green laser line from both sides, no matter how much it is deflected. The water tunnel has a partition for the forced flow of water upside down in one direction and downwards in the opposite direction. A variable fluid velocity external water pump can be used to experiment with different fluid velocities, and propulsion system characteristics can be accomplished in a closed-loop control system, as depicted in the paper^26^. However, at this point in the investigation, the number of parameters impacting fin characteristics was reduced, and the measurement was performed without using an external water pump.

### Mechanical parameters of the fins

It is worth mentioning that both the thrust and the drag forces depend on the material of the fin and the control parameters. Therefore, a series of measurements were performed with direct measurements of the fin-fluid interaction using the force sensor, as shown in Fig. 7. Based on the research presented in the paper^19^ for the test, one fin was adopted with length 150 mm, width 55 mm and thickness 0.75 mm. The fin was made from polymethyl methacrylate with the next parameters:

- density: 1.18 kg/m^3^;
- tensile strength: 70 MPa;
- flexural modulus: 2.9 GPa.

Thanks to the shatter resistance, it can be used for many cycles of fin oscillations. It also provides outstanding stability against environmental conditions. Further, their transparency is convenient for the Particle Image Velocimetry (PIV) technique, and the laser can highlight both sides of the fin no matter how it is deflected (see Fig. 7).

### Servomechanism and control parameters

The fins in BUUV are driven by a servomechanism dynamixel AX-12+ (http://www.dynamixel.com/) mounted inside the hull. The significant characteristics of the Dynamixel AX-12+ are stall torque of 1.5 Nm (at 12 V, 1.5 A). The fin oscillation frequency depends on the rotational velocity of the servomechanism as well as the tilt angle of the fin. All the parameters are tabulated in the following section. The embedded control function was used to remote calculate electric energy consumption by the servomotor. The power input to the servomotor was calculated as a product of the measured voltages (*U*) and the electric current (*I*) across the servomotor. Although the wide area of the fin angle of attack and the fin frequency oscillations were implemented, just a few chosen results are discussed here.

Algorithm 1 outlines an open-loop control approach for a servo mechanism driving a fin, incorporating real-time power consumption monitoring. The control algorithm continuously adjusts the fin’s position based on a predefined oscillatory motion while measuring the electrical current drawn by the motor. Using these measurements, the algorithm calculates the instantaneous power consumption, providing data for system efficiency analysis. This implementation enables the assessment of energy usage and can be utilized to optimize power efficiency in autonomous marine vehicles.

**Algorithm 1:**
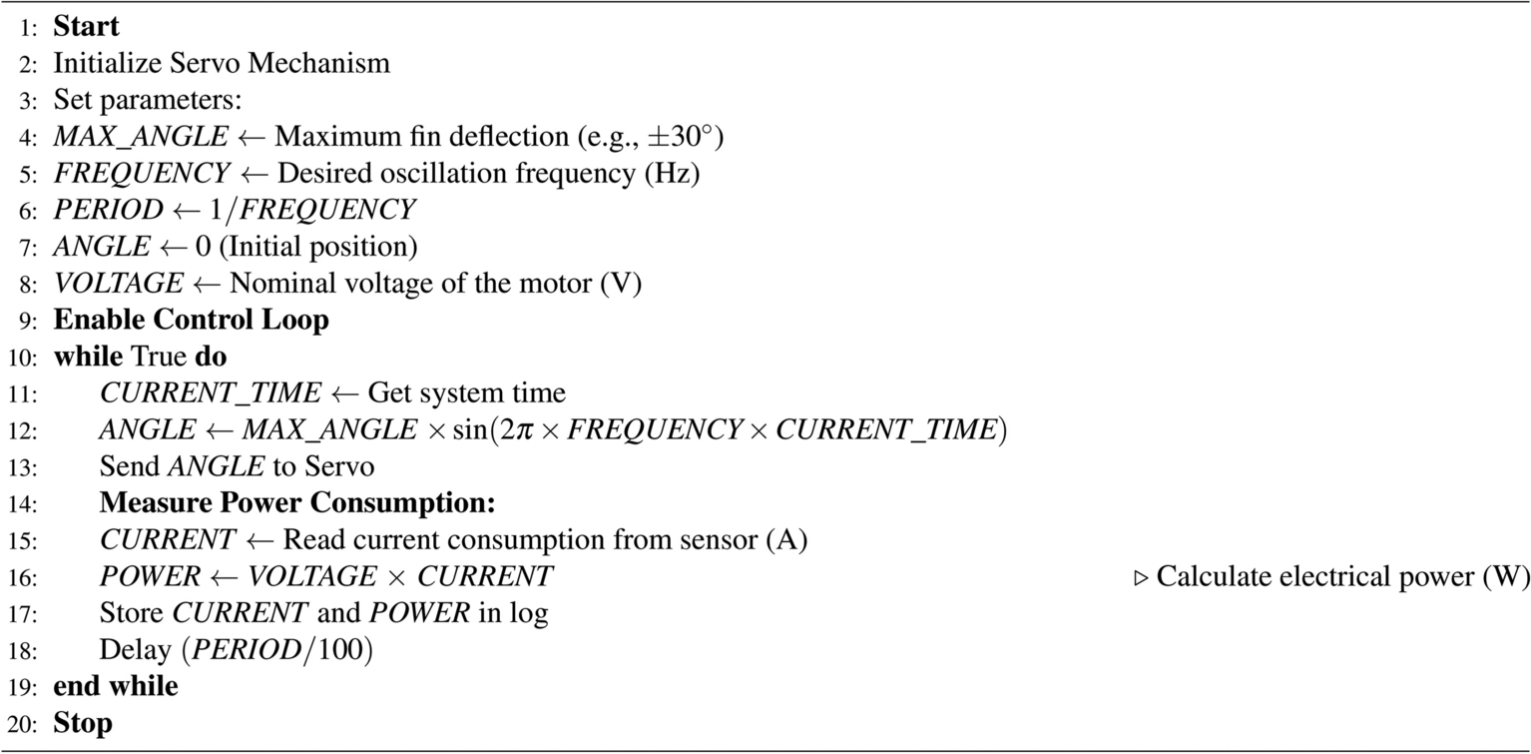
Open-loop control of a servo mechanism driving a fin with power monitoring

Since the shape of the hull causes turbulence in the area of influence of the tail fins, this study analyses only the parameters of the side fins. The artificial fin for BUUV has an open-loop control system that does not provide information about fin deflection or the amplitude of a trailing edge. Considering the above, this paper provides an analysis based on the measurements made in the water tunnel.

For thrust measurements, the vehicle is directly attached to the force sensor (https://www.axis.pl/en/fb/392-fb5.html, denoted by the number 3 in Fig. 7a). To reduce friction, ball bearings (numbered 4 in Fig. 7a) were used to direct trust transfer created by BUUV fins (numbered 2 in Fig. 7a) in interface with fluid. The force sensor parameters were set at a range of 5 N, a sampling time of 0.01 s, and a resolution of 0.1 mN. The system propulsion efficiency was calculated based on the measurements provided, as depicted in the next section.

### Particle image velocimetry (PIV) method

Particle Image Velocimetry (PIV) is a widely used optical technique for measuring velocity fields in fluid flows. Its non-intrusive method enables the measurement of instantaneous velocity fields with high spatial and temporal resolution, making it a valuable tool for characterizing fluid flow in complex geometries and studying flow behaviour. For this study, the PIVLab software was utilized^27^ to analyze the interaction between the fin and the fluid.

**Fig. 8:**
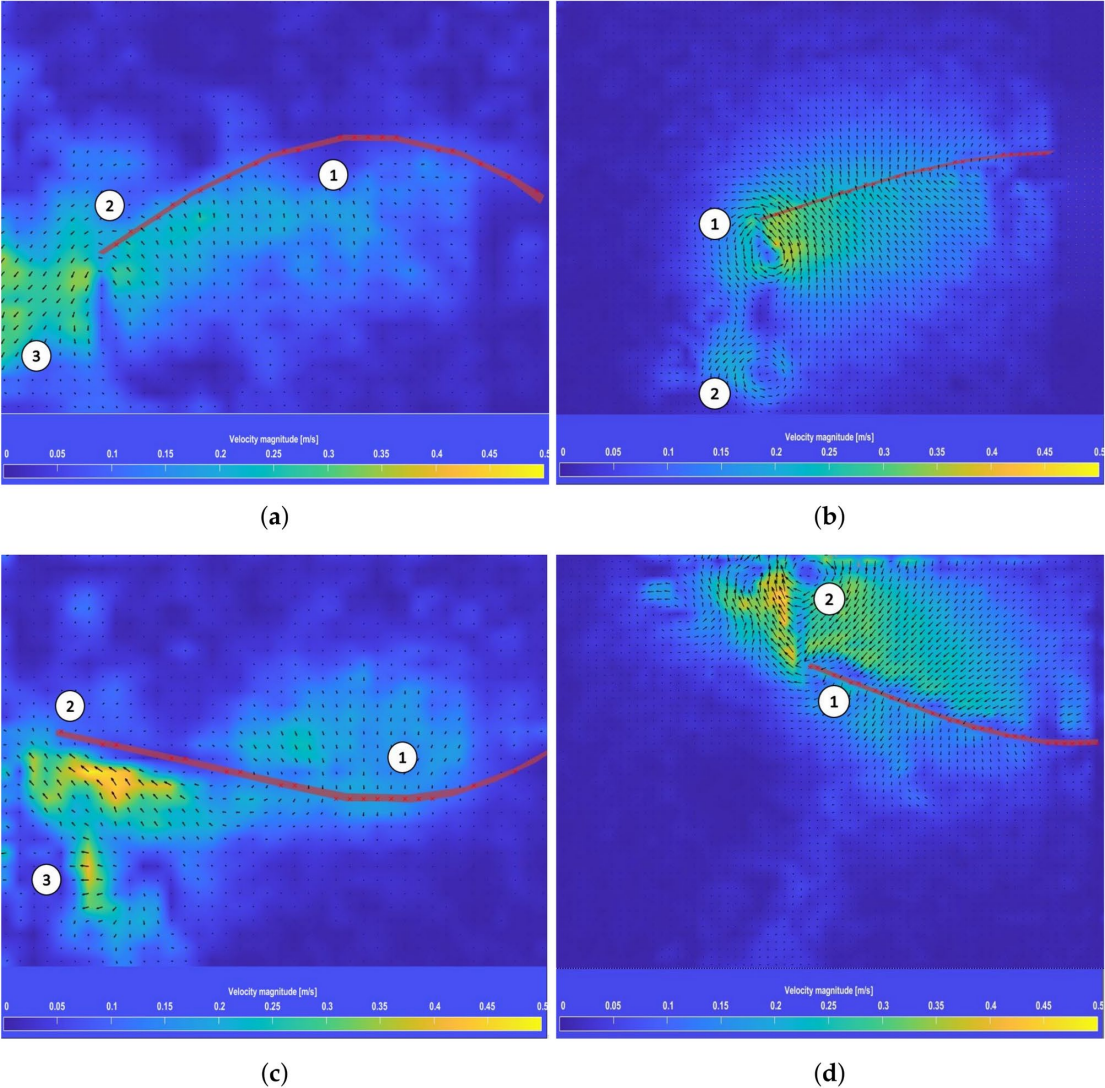
Velocity vectors in the area of interaction between fluid and the fin length: 250 mm when moving the fin up (**a**) and down (**c**), 150 mm when moving the fin up (**b**) and down (**d**)^19^.

The experiments used markers with neutral buoyancy and small dimensions (diameter 10 $\mu$m), which did not disrupt the natural flow dynamics. These markers, composed of transparent glass balls surrounded by silver, ensured high reflectivity when illuminated by a 1 *W* green linear laser. This setup enabled the high-speed camera (HSC) to capture detailed velocity fields. The PIV analysis was conducted for an illuminated plane perpendicular to the side fin, as depicted in Fig. 8.

The analysis focused on calculating fluid velocity based on the average values of velocity vectors within the interaction region between the fins and the fluid. These tests used fins with dimensions of 55 mm width, 0.75 mm thickness, and lengths of 150 mm and 250 mm. Results for the longer fins (250 mm) during upward and downward movement are shown in Fig. 8a,c, while results for the shorter fins (150 mm) are shown in Fig. 8b,d.

The velocity fields revealed distinct flow characteristics based on fin length and movement. For shorter fins (150 mm), a larger vertical flow area was observed, but significant turbulence, especially at the fin tips (marked as region 1 in Fig. 8b), was present. In contrast, longer fins (250 mm) generated more laminar flow near the tips due to their flexibility, as seen in regions marked 2 in Fig. 8a,c. Regions 3 in Fig. 8a,c illustrate how the middle section of the longer fins initiates water movement, creating preliminary velocity vectors further enhanced by the trailing edge. This sequential process results in velocity vectors forming in a reverse direction relative to BUUV movement.

Turbulence, particularly at the trailing edge and fin tips, was a significant energy dissipation source. The results highlight the importance of minimizing turbulence to improve energy efficiency. For instance, turbulence observed in shorter fins at both the trailing edge and additional regions (numbered 2 in Fig. 8b,d) underscores the need for careful design optimization.

The variations in fin length, as presented in^19^, impacted several non-dimensional parameters, including the shape factor, stiffness coefficient, Reynolds number, and consequently, the Strouhal number. Although the Strouhal number offers a basis for comparing the energy efficiency of artificial fins to marine animals, direct thrust measurements presented in the following section provide a more practical evaluation of propulsion performance.

## Propulsion system efficiency

The efficiency of a biomimetic propulsion system for an unmanned underwater vehicle depends on several environmental and hull design parameters, including propulsion system configuration and control algorithm. As the drag force increases as a square function of the vehicle, it determines the amount of energy required to maintain a desired velocity.

Taking into consideration the propulsion efficiency ($\eta$), it can be defined as a ratio of thrust coefficient $C_{T}$ to the input power coefficient $C_{P};$

1 $$\begin{aligned} \eta = \frac{C_{T}}{C_{P}} \end{aligned}$$

where:

2 $$\begin{aligned} C_{T} = \frac{T}{0.5\rho u^{2} cL} \end{aligned}$$

and:

3 $$\begin{aligned} C_{P} = \frac{P}{0.5\rho u^{3} cL} \end{aligned}$$

*u*—the fluid velocity; $\rho$—the fluid density; *c*, *L*—fin dimensions (length, width); *P*—electric power delivered to the servomechanism; *T*—thrust generated by the fin;Both coefficients ($C_{T}, C_{P}$) cannot be measured directly or calculated due to thrust ambiguity for flexible fins as depicted in the paper^28^. The thrust cannot be separated from the drag force due to the flexible fins’ simultaneous generation of drag and thrust. The performance of the propulsion system^29^ can be estimated as a difference between the thrust and the drag force (4).

4 $$\begin{aligned} T = T_N + T_D \end{aligned}$$

where: *T*—the net thrust; $T_N$—the thrust; $T_D$—the drag force.The efficiency of a biomimetic propulsion system for an unmanned underwater vehicle is a complex function of several parameters, and optimizing its performance requires careful consideration of the interplay between these factors. Further research was carried out in the water tunnel, taking into account the greater reliability of the results from experimental tests carried out in water tunnels compared to simulation tests. The Particle Image Velocimetry (PIV) method was used to analyse fluid-structure interaction (FSI) phenomena in detail. Details related to the measurement results are described in the next section.

## Experimental results

Considering the oscillation frequency’s impact on the net thrust generation and the propulsion system energy efficiency, the servomechanism’s non-linear characteristics were excluded. This was done by measuring electric energy delivered to the servomechanism when working without additional load. The results are presented in Table 1 for different fin angle deflection and different frequencies of oscillation. The servomechanism operated at a constant angular velocity for various deflection angles, which were symmetrical for upward and downward movements. The oscillation frequency was adjusted by modifying the angular velocity of the servomechanism.

Then, the same control parameters were provided for servomechanism working when submerged in water (see Table 2). The differences between the servomechanism working in the air and water for different angles of deflection and frequencies of oscillations are presented in Table  3. This method was implemented to exclude the nonlinear servomechanism efficiency characteristic, which could provide additional, undesired inaccuracies during the biomimetic propulsion system efficiency analysis. During the energy efficiency analysis, the net thrust generated by the propulsion system was directly measured, and the results are presented in Table 4. The energy efficiency calculated as the ratio of net thrust generated by the propulsion system to the electric energy delivered to the servomechanism was calculated and included in Table 5.

**Table 1:** Average value of the servomechanism load with no water resistance.

f Hz	α			
	45°	40°	35°	30°
4	4.93 W	4.91 W	4.85 W	4.80 W
3	4.75 W	4.71 W	4.63 W	4.52 W
2	3.58 W	3.31 W	3.24 W	3.21 W
1	1.08 W	1.08 W	1.07 W	1.06 W

**Table 2:** Average value of the servomechanism load with water resistance.

f Hz	α			
	45°	40°	35°	30°
4	10.41 W	9.10 W	8.77 W	8.6 W
3	9.89 W	8.83 W	8.7 W	8.03 W
2	6.12 W	5.06 W	4.92 W	4.87 W
1	1.68 W	1.51 W	1.36 W	1.34 W

**Table 3:** The net value of the servomechanism load calculated as a difference between loaded and unloaded servo.

f Hz	α			
	45°	40°	35°	30°
4	5.47 W	4.19 W	3.91 W	3.80 W
3	5.14 W	4.12 W	4.07 W	3.51 W
2	2.54 W	1.75 W	1.67 W	1.66 W
1	0.60 W	0.43 W	0.29 W	0.28 W

**Table 4:** The mean value of the net trust.

T N	α			
f Hz	45°	40°	35°	30°
4	0.27	0.28	0.26	0.21
3	0.30	0.30	0.27	0.22
2	0.19	0.19	0.18	0.18
1	0.08	0.08	0.08	0.07

**Table 5:** Energy efficiency.

f Hz	α			
	45° (%)	40° (%)	35° (%)	30° (%)
4	4.96	6.57	6.52	5.6
3	5.76	7.31	6.61	6.39
2	7.32	10.7	10.99	10.68
1	12.83	18.88	26.34	26.35

**Fig. 9:**
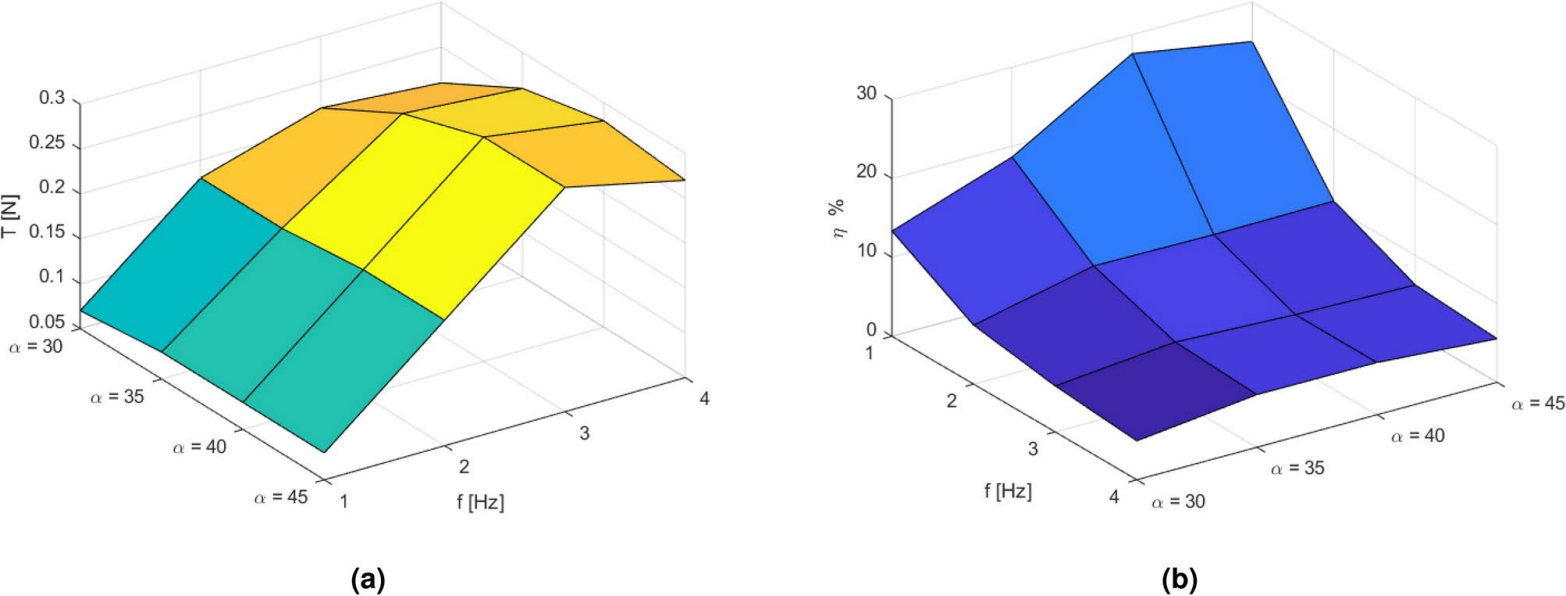
The measured results of the net thrust (**a**) and the biomimetic propulsion system efficiency (**b**) [source: own study].

For better analysis, the results are also presented in Fig. 9, where the net thrust is depicted in Fig. 9a, the energy efficiency is presented in Fig. 9b. When analysing the results, it can be seen that the maximal net thrust is achieved for oscillation equal to 3 Hz and the deflection angle $\alpha = 40^{\circ}$ (see Fig. 9a). It can also be observed that when the net thrust increases, the energy efficiency decreases (see Fig.  9b), which is observed in marine organisms when they swim freely and when they run away from danger.

## Methodology outline

The energy efficiency of artificial fish depends on several factors, including their design, size, and materials used in their construction. Some factors that can affect the energy efficiency of artificial fish include the choice of propulsion system and the ability to reduce drag and turbulence in the water.

For analysis of the efficiency of biomimetic propulsion systems, the following actions are recommended:

1. Definition and specification of the hull type of vehicle and the assumed value of thrust needed;
2. Take into consideration the actuator characteristics;
3. Check the material parameters as depicted in the paper^18^;
4. Identify the kinematics parameters as described in the paper^26^;
5. Measure the Fluid-Structure Interaction (FSI) and the net thrust generated with the electric energy consumption (see paper^19^);
6. Analyze the turbulence area of interest and the region energy dissipation based on the PIV method (see paper^19^);
7. Calculate the energy efficiency as delivered in this paper.
8. Choose the best ratio of the generated thrust to the energy efficiency and adapt it to the propulsion control system.
9. Implement the control algorithm for the desired net thrust value concerning the energy efficiency characteristic.

## Innovativeness

The innovativeness of the research conducted on the single-fin biomimetic propulsion system lies in the measurement and analysis of a full working cycle of the kinematic parameters, the generated net thrust, the electric energy consumed by the actuator system, and the velocity vectors and their components in the interaction area between the fin and the fluid. The proposed methodology enables a detailed analysis of the interaction between the biomimetic propulsion system and the surrounding fluid, facilitating the selection of optimal structural and control parameters.

## Future research

In future research, we plan an in-depth optimization process leveraging Fluid-Structure Interaction (FSI) analysis. We aim to integrate experimental data with AI-driven optimization to analyze turbulence effects on biomimetic propulsion efficiency. This research will focus on simulations based on Physics-Informed Neural Networks (PINNs) to model and predict the interaction between flexible fins and turbulent flows.

Furthermore, we will utilize an intelligent water tunnel operating in an autonomous mode, enabling real-time testing and adaptive fin adjustments based on experimental flow data. This approach will facilitate the development of advanced control strategies aimed at enhancing propulsion efficiency while minimizing hydrodynamic disturbances.

## Data Availability

The datasets used and/or analysed during the current study are available from the corresponding author on reasonable request.
